# Molecular genetic analysis of a cattle population to reconstitute the extinct *Algarvia *breed

**DOI:** 10.1186/1297-9686-42-18

**Published:** 2010-06-11

**Authors:** Catarina Ginja, Maria CT Penedo, Maria F Sobral, José Matos, Carla Borges, Dina Neves, Teresa Rangel-Figueiredo, Alfredo Cravador

**Affiliations:** 1 University of California, Veterinary Genetics Laboratory, One Shields Avenue, DAVIS, California 95616, USA; 2 Direcção Geral de Veterinária-DSPA, Rua Elias Garcia 30, Venda Nova, 2704-507 AMADORA, Portugal; 3 L-INIA - Pólo do Lumiar Unidade de Investigação em Recursos Genéticos, Ecofisiologia e Melhoramento de Plantas, Grupo de Biologia Molecular, Estrada do Paço do Lumiar, 22 Ed S 1º andar 1649-038 LISBOA, Portugal; 4 Universidade do Algarve, FCT, Campus de Gambelas, 8005-139 FARO, Portugal; 5 CECAV - Universidade de Trás-os-Montes e Alto Douro, Departamento de Zootecnia, Apartado 1013, 5000-911 VILA REAL, Portugal; 6 IBB/CGB - Universidade do Algarve, Campus de Gambelas, 8005-139 FARO Portugal

## Abstract

**Background:**

Decisions to initiate conservation programmes need to account for extant variability, diversity loss and cultural and economic aspects. Molecular markers were used to investigate if putative Algarvia animals could be identified for use as progenitors in a breeding programme to recover this nearly extinct breed.

**Methods:**

46 individuals phenotypically representative of Algarvia cattle were genotyped for 27 microsatellite loci and compared with 11 Portuguese autochthonous and three imported breeds. Genetic distances and factorial correspondence analyses (FCA) were performed to investigate the relationship among Algarvia and related breeds. Assignment tests were done to identify representative individuals of the breed. Y chromosome and mtDNA analyses were used to further characterize Algarvia animals. Gene- and allelic-based conservation analyses were used to determine breed contributions to overall genetic diversity.

**Results:**

Genetic distance and FCA results confirmed the close relationship between Algarvia and southern Portuguese breeds. Assignment tests without breed information classified 17 Algarvia animals in this cluster with a high probability (q > 0.95). With breed information, 30 cows and three bulls were identified (q > 0.95) that could be used to reconstitute the Algarvia breed. Molecular and morphological results were concordant. These animals showed intermediate levels of genetic diversity (MNA = 6.0 ± 1.6, R_t_ = 5.7 ± 1.4, H_o_ = 0.63 ± 0.19 and H_e_ = 0.69 ± 0.10) relative to other Portuguese breeds. Evidence of inbreeding was also detected (F_is_ = 0.083, *P* < 0.001). The four Algarvia bulls had Y-haplotypes H6Y2 and H11Y2, common in Portuguese cattle. The mtDNA composition showed prevalence of T3 matrilines and presence of the African-derived T1a haplogroup. This analysis confirmed the genetic proximity of Algarvia and Garvonesa breeds (F_st_ = 0.028, *P* > 0.05). Algarvia cattle provide an intermediate contribution (CB = 6.18, CW = -0.06 and D1 = 0.50) to the overall gene diversity of Portuguese cattle. Algarvia and seven other autochthonous breeds made no contribution to the overall allelic diversity.

**Conclusions:**

Molecular analyses complemented previous morphological findings to identify 33 animals that can be considered remnants of the Algarvia breed. Results of genetic diversity and conservation analyses provide objective information to establish a management program to reconstitute the Algarvia breed.

## Background

Breeding practices designed to alleviate production constraints are prejudicial to the survival of traditional domestic animal breeds, and tend to lead to impoverishment of the gene pool [1]. The Food and Agriculture Organization of the United Nations has encouraged a series of conservation measures designed to help prevent irreversible loss of diversity of domesticated animal species [2]. A heightened awareness of the cultural, historical and social heritage represented by traditional breeds has led to increased interest in their preservation [3]. Despite its small geographic area, Portugal hosts a wide variety of domestic breeds [4], with as many as 13 autochthonous cattle breeds recognized [4,5]. Analysis of genetic diversity of some of these breeds has used blood protein polymorphisms [6], microsatellite variation [7-12], mitochondrial DNA (mtDNA) [13,14] and Y chromosome sequence variation [15]. These studies have shown that, among European cattle, the Mirandesa breed is one of the most important targets for preservation based on its contribution to diversity [8]. In general, southern European cattle appear to represent particularly important reservoirs of genetic diversity [16].

*Algarvia *cattle are native to the Algarve region of southern Portugal and were first represented in 1868 [17] and later described with more details [18-23]. Based on its morphology, the breed has been classified in the Aquitanian (or Red Convex) group, together with Alentejana, Mertolenga, Garvonesa and Minhota [23,24]. Except for Minhota, these breeds are distributed throughout the southern river Tagus valley. *Algarvia *cattle were used predominantly for meat production and/or draft. Although they were never formally registered as an independent breed, 25,000 - 29,000 animals were officially catalogued between 1940 and 1970 [25]. Since then, the population has declined rapidly and the breed was considered to be effectively extinct by the 1980s [1], an event that subjected the Regional Agricultural Authorities to criticism for failure to implement proper conservation measures.

The morphology of eight autochthonous Portuguese cattle breeds has been analyzed by numerical taxonomic methods [26], and these analyses have been extended recently to include animals thought to derive from the *Algarvia *breed [27]. This study identified a uniform group of animals that shared many of the phenotypic characteristics of that breed. These remnants were preserved mainly for cultural reasons by traditional breeders in different areas of Algarve and southern Alentejo regions.

In the present study, we have characterized these putative *Algarvia *animals using nuclear microsatellites, Y chromosome markers and mtDNA sequences to determine to which extent they can be genetically distinguished from related autochthonous breeds, and to identify individuals that would represent suitable progenitors for a breeding programme to reconstitute the *Algarvia *breed.

## Methods

### Sampling procedure

Forty-six animals (42 females and four males) were chosen from ten independent herds and consisted of individuals thought to derive from the *Algarvia *cattle on the basis of phenotypic similarity, as judged by traditional farmers with experience on the breed characteristics [27].

Additionally, samples from Garvonesa (29) and Preta (47) breeds were collected because they are present throughout the southern region of the country and admixture with *Algarvia *cannot be excluded. The herds in which putative *Algarvia *individuals were located also had animals from the Alentejana breed. A 9 ml whole blood sample was collected from each individual by jugular venipuncture in tubes containing EDTA-K3 as anticoagulant. Genomic DNA was extracted from leukocytes using the Puregene DNA Isolation Kit (Gentra Systems, Minneapolis, USA).

### Microsatellite genotyping

A set of 27 microsatellite markers *(BM1818, BM1824, BM203, BM2113, BM2613, BRRIBO, CSSM36, CYP21, ETH10, ETH152, ETH225, ETH3, HEL11, HEL13, HEL9, ILSTS035, ILSTS065, INRA023, MGTG4B, RM006, RM067, SPS115, TGLA122, TGLA126, TGLA227, TGLA345 *and *TGLA53*) was used to genotype each animal. Multiplex PCR were performed using fluorescence-labelled primers, as described by Mateus *et al*. [12]. PCR products were separated by capillary electrophoresis on ABI PRISM^® ^310 instruments (Applied Biosystems, Foster City, CA) and fragment size analysis was done with STRand software [28]. Genotypes from nine Portuguese autochthonous and three imported breeds were obtained from Mateus *et al*. [12,29], thus the complete dataset included, in addition to the above breeds, Alentejana (50 individuals), Arouquesa (50), Barrosã (50), Brava de Lide (40), Minhota (50), Marinhoa (51), Maronesa (47), Mertolenga (50) and Mirandesa (50), Charolais (45), Friesian (35) and Limousin (48). Reference samples were included in all PCR assays to standardize allele sizing across datasets.

### Statistical analysis of microsatellite data

Allele frequencies were determined with GENALEX version 6 [30]. The software GENEPOP version 3.4 [31] was used to perform global and per locus/per population Hardy-Weinberg Equilibrium (HWE) tests, and to test for genotypic linkage disequilibrium (LD). Exact probability tests were done for loci with four or fewer alleles; otherwise, a Markov chain method was employed [31] with 10,000 dememorization steps, 500 batches and 5,000 iterations. GENETIX version 4.02 [32] was used to estimate within-population observed (H_o_) and unbiased expected (H_e_) heterozygosities [33], the mean number of alleles (MNA), and inbreeding coefficients (F_is_) [34]. The statistical significance of F_is _> 0 was obtained based on 1,000 permutations. Pairwise population F_st _values were calculated with FSTAT version 2.9.3 [35] and *P*-values obtained based on 1,000 randomizations. This software was also used to estimate allelic richness (R_t_) per locus and population. To investigate breed relationships, neighbour-joining (N-J) dendrograms [36] were constructed from D_A _genetic distances [37] using POPULATIONS version 1.2.28 [38]. Bootstrap values were obtained with 1,000 replicates over loci. A dendrogram based on allele-sharing distances between individuals was also constructed using this software. TREEVIEW version 1.6.6 [39] was used to visualize and edit the dendrograms. A factorial correspondence analysis (FCA) was done with GENETIX to investigate relationships among individuals. Assignment tests were performed to identify individuals most representative of the *Algarvia *breed and to detect admixture. STRUCTURE version 2.2 [40] was used to estimate the most probable number of population clusters (K). The analysis was done without prior information on populations, assuming correlated allele frequencies and admixture [41]. Ten independent runs with 100,000 Markov Chain Monte Carlo (MCMC) iterations and 10,000 *burn-in *were performed at each K (1 ≤ K ≤ 9) to calculate ΔK as in Evanno *et al*. [42]. A longer run (1,000,000 iterations and 100,000 *burn-in*) was done for the most probable K to determine the number of individuals within each cluster. An assignment test with these settings but including prior breed information was also performed. The partially Bayesian simulation-exclusion procedures of GENECLASS version 2.0 [43] were used for assignment tests with 10,000 Monte Carlo resamplings of individuals [44-46].

The contribution of each population to the overall genetic diversity was analysed considering the within- (CW) and between-breed (CB) diversity components, and aggregate genetic diversity (D1 = F_st_*CB + (1-F_st_)*CW) as described by Ollivier and Foulley [47]. METAPOP software version 1.0.2. [48] was used to account for allelic diversity and estimate the contribution of each population (c_i_) to a pool of maximal genetic diversity [49]. Equal weights were given to within- and between-breed coancestries (λ = l). The average molecular coancestry (*f*_m_) of each population was also obtained with this software.

### Analysis of Y chromosome markers

The four putative *Algarvia *males were genotyped for one SNP (*UTY intron 19 AY936543: g.423C > A*), one *indel *(*ZFY intron 10 AF241271: g.697_8indelGT*), and five microsatellites (*DDX3Y_1, BM861, INRA189, UMN0103 *and *UMN0307*) located in the male-specific region of the bovine Y chromosome. Analyses were done as described by Ginja *et al*. [15] for a comparison with previously identified patrilines in Portuguese autochthonous breeds.

### Analysis of mtDNA sequence variation

A 919 bp PCR fragment containing the complete mtDNA control region was obtained and sequenced for *Algarvia *animals. The analysis was done as described by Ginja *et al*. [50] and sequences were aligned with the taurine reference sequence [GenBank: V00654, [51]] using the Multalin interface http://bioinfo.genotoul.fr/multalin/multalin.html. Haplotypes were identified with GENALEX [30] and ARLEQUIN version 2.0 [52] was used to calculate haplotype diversity (H), nucleotide diversity (π), and the mean number of pairwise nucleotide differences (MNPD) accounting for heterogeneity of substitution rates per site [53]. mtDNA sequences of the southern Portuguese autochthonous breeds and the imported Limousin were obtained from the GenBank database [accession numbers: FJ815445-59, FJ815525-40, FJ815573-88, FJ815620-35 and FJ815880-95] and used in ARLEQUIN to estimate pairwise-population F_st _values (5% significance level obtained with 10,000 permutations). A Median-Joining (MJ) network of haplotypes was constructed with NETWORK version 4.5.10 [54] software to investigate breed relationships.

## Results

### Genetic relationships between *Algarvia *and other breeds

**Figure 1:**
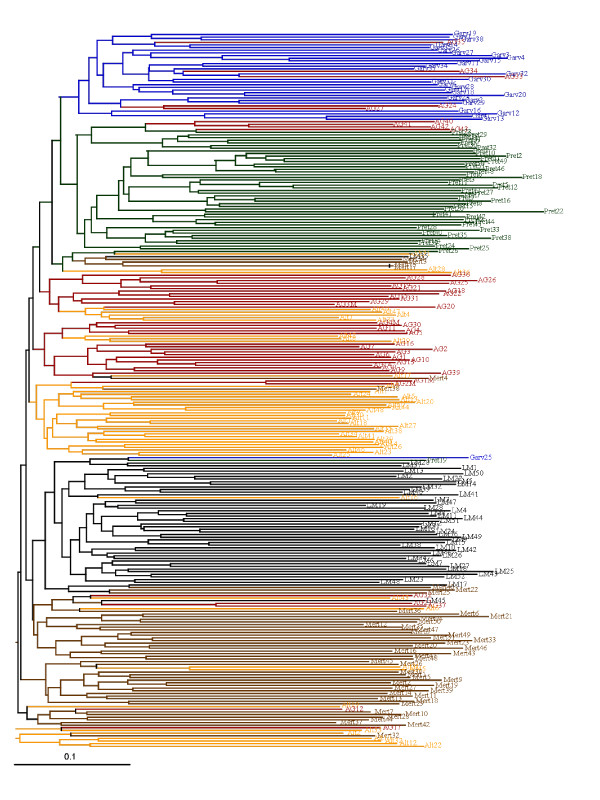
**Neighbour-Joining dendrogram**. The N-J dendrogram is based on allele sharing distances among animals from the autochthonous breeds Alentejana (Alt, N = 50), Garvonesa (Garv, N = 29), Mertolenga (Mert, N = 50), Preta (Pret, N = 47), the *Algarvia *population (AG, N = 46) and the imported Limousin(LM, N = 48)

The genetic distance analysis confirmed that the putative *Algarvia *animals were close to the southern Garvonesa and Alentejana breeds, and distant from northern breeds such as Mirandesa (Additional file 1 Figure S1). It also showed that this group of animals was more closely related to Preta than to Brava de Lide, both of which are considered to belong to the Black Orthoid breed group. Relationships among the individuals of *Algarvia *and of southern Portuguese breeds that clustered with *Algarvia *in Additional file 1 Figure S1 are shown in the N-J dendrogram of allele sharing distances in Figure 1. Limousin cattle was included in the analysis because they are raised in the southern region of Portugal and have been used to upgrade the autochthonous breeds [24]. Thirty-two *Algarvia *animals formed two closely related subgroups each containing a few Alentejana animals. Five *Algarvia *individuals clustered with Garvonesa (AG24, AG27, AG33, AG34 and AG35), four with Preta (AG40, AG41, AG42 and AG43) and five with Mertolenga (AG12, AG17, AG23, AG32 and AG37). Results of the FCA showed that Preta is the most distant breed but that among southern Portuguese breeds including *Algarvia *genetic differentiation was weak (Additional file 2 Figure S2). The relationships among individuals represented in the FCA graph were consistent with those shown in Figure 1.

### Assignment of *Algarvia *cattle

**Figure 2:**
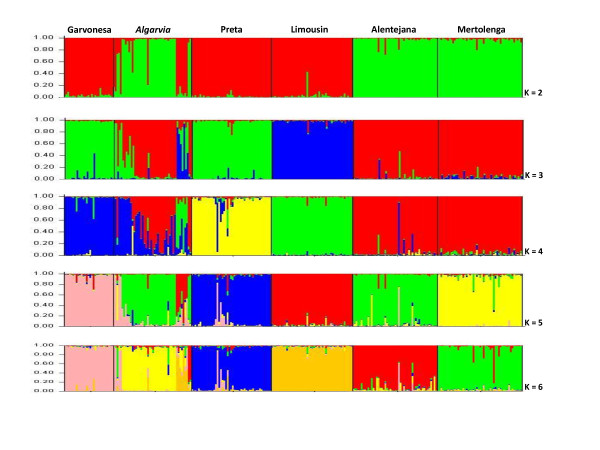
**Graphical representation of the estimated membership coefficients (q)**. STRUCTURE was used to obtain q values for each individual of the southern Portuguese breeds and the imported Limousin with K varying from 2 to 6

STRUCTURE analyses assume that within a population all loci are in HWE and linkage equilibrium [40]. Although for some breeds a high number of loci showed significant (*P *< 0.05) deviations from HWE without correction for multiple testing (Additional file 3 Table S1), the assignments with STRUCTURE were conducted to include all loci, because some deviations from HWE are not expected to affect the performance of the test [55]. The HWE deviations found in Brava de Lide and Preta breeds are most probably related with a *Wahlund *effect and/or inbreeding, considering that the F_is _values for these breeds were highly significant [see discussion for Brava de Lide in [12]]. Within breeds, LD was significant (*P *< 0.001) for one pair of loci in the Alentejana breed, four in the Preta and four in the Brava de Lide, but none of these corresponded to markers located on the same chromosome. The assignment tests of STRUCTURE and GENECLASS were done exclusively for the *Algarvia *animals, the related southern breeds (Alentejana, Garvonesa, Mertolenga and Preta) and the Limousin cattle to determine which *Algarvia *animals clustered as an independent group, and to detect admixture. The STRUCTURE assignments without prior information on source breeds showed the highest ΔK at K = 6 (Additional file 4 Figure S3). The estimated genotype membership coefficients (q) obtained for each individual are shown in Figure 2. *Algarvia *animals clustered with the Alentejana and Mertolenga breeds at K = 2 and only appeared as an independent cluster at K = 6.

Approximately 54% of all individuals were correctly allocated to each of the six clusters with q ≥ 0.95 (Table 1). For the *Algarvia *cluster (K = 6), the average individual genotype membership proportion (Q) was 0.66 ± 0.40 and this cluster contained 17 individuals (37%) with q ≥ 0.95 (AG4, AG5, AG6, AG7, AG8, AG10, AG13, AG14, AG15, AG16, AG18, AG19, AG22, AG24, AG25, AG27 and AG28). Among the remaining *Algarvia *animals, one (~2%) was misclassified with q ≥ 0.95 and 28 (~61%) were not assigned to this population (q < 0.95). When prior information on populations was used, ~85% of all individuals were correctly allocated with q ≥ 0.95 (Table 1). Among the 46 *Algarvia *animals, 33 (72%) were allocated to this cluster with q ≥ 0.95. These included the 17 listed above plus AG1, AG2, AG2M, AG3M, AG4M, AG9, AG11, AG20, AG21, AG26, AG29, AG30, AG31, AG40, AG42 and AG43. Eleven (24%) *Algarvia *animals were not assigned to this population (q < 0.80) and admixture was detected with Garvonesa (AG33, AG34, AG35 and AG36), Limousin (AG23, AG32, AG37, AG39 and AG41) and Mertolenga breeds (AG12 and AG17). For the *Algarvia *population, the average value of Q was 0.85 ± 0.27 and was the lowest among all breeds.

**Table 1:** Results of the Bayesian assignment tests done with STRUCTURE

		Without prior information			With prior information		
			
Population	N	Q ± SD	**% Corr. assign**.	**% Mis**.	Q ± SD	**% Corr. assign**.	**% Adm**.
Alentejana	50	0.89 ± 0.14	48.0	0.0	0.97 ± 0.05	82.0	2.0
***Algarvia***	**46**	**0.66 ± 0.40**	**37.0**	**2.2**	**0.85 ± 0.27**	**71.7**	**23.9**
Garvonesa	29	0.94 ± 0.07	69.0	0.0	0.97 ± 0.07	89.7	3.4
Limousin	48	0.95 ± 0.05	70.8	0.0	0.98 ± 0.04	93.8	2.1
Mertolenga	50	0.90 ± 0.15	50.0	0.0	0.97 ± 0.07	88.0	4.0
Preta	47	0.89 ± 0.18	53.2	0.0	0.94 ± 0.19	85.1	7.4
Overall	270	0.87 ± 0.22	53.7	0.4	0.95 ± 0.15	84.8	8.5

N: sample size; Q: average genotype membership proportions; SD: standard deviation; Corr. assign.: percentage of correctly assigned animals with q ≥ 0.95; Mis.: percentage of misassigned animals with q ≥ 0.95; Adm.: percentage of admixed animals with q < 0.800

Results of GENECLASS assignments are summarized in Additional file 5 Table S2. Animals were correctly assigned if the genotype probability was higher than the threshold exclusively in their source population. For *Algarvia *animals, ~22 to 50% of the individuals were correctly assigned with accuracies > 0.94 (ratio between the number of correctly assigned individuals and the sum of correctly and incorrectly assigned). Depending on the threshold considered, between 2 and 20% of the *Algarvia *animals were excluded (genotype probabilities lower than the threshold in all populations) and 30 to 76% were assigned to multiple populations (genotype probabilities greater than the threshold in at least two populations).

### Genetic diversity of *Algarvia*

The genetic diversity (MNA = 6.0 ± 1.6, R_t _= 5.7 ± 1.4, H_o _= 0.63 ± 0.19 and H_e _= 0.69 ± 0.10) of the 33 *Algarvia *animals identified with STRUCTURE was identical to that found for the related Alentejana breed, and slightly lower than the average estimates across all breeds included in this study (Additional file 3 Table S1, MNA = 6.8 ± 0.6, R_t _= 6.0 ± 1.8, H_o _= 0.67 ± 0.05 and H_e _= 0.70 ± 0.03). Ten population-specific alleles were found in the *Algarvia *population but none had a frequency greater than 0.05. Deviations from HWE were significant (*P *< 0.05) due to heterozygote deficit at ten loci (*BM203, BM1824, BM2113, BRRIBO, ETH152, ILSTS035, SPS115, TGLA53, TGLA122*, and *TGLA345*). Evidence of inbreeding within the *Algarvia *group could be inferred from the F_is _estimate (0.083) which was significantly (*P *< 0.001) greater than zero. The molecular coancestry of *Algarvia *animals (*f*_m _= 0.322) was slightly higher than the overall value of 0.310. Pairwise population F_st _estimates showed that *Algarvia *animals are genetically closer to the Alentejana breed (F_st _= 0.045, *P <*0.05) than to the other southern breeds (results not shown).

### Y chromosome haplotypes of *Algarvia*

Among the four *Algarvia *bulls, two (ALG3M and ALG4M) had the H11Y2 haplotype which is fixed in the Alentejana breed but also common in other Portuguese breeds, whereas the two other animals (ALG1M and ALG2M) had the H6Y2 haplotype which is fixed in the Garvonesa breed but also found in other Portuguese breeds as well as in Charolais and Limousin breeds [15].

### mtDNA haplotypes of *Algarvia*

Complete mtDNA control region sequences (909 bp) were obtained for the 33 *Algarvia *animals (four bulls and 29 females) assigned to this group with STRUCTURE (sequence quality of AG19 was low and thus was discarded). Sequence alignment is shown in Additional file 6 Figure S4 with polymorphic positions represented. A total of 12 distinct haplotypes was identified [GenBank: G086285-G086317] based on 21 variable sites, of which 11 were phylogenetically informative, nine were singletons, and one was an *indel*. The European T3 haplogroup (T at nt16255) was the most frequent (29 animals) but the African-derived T1a type (T at nt16050, C at nt16113 and C at nt16255) was also detected in three *Algarvia *individuals (AG16, AG40 and AG43). Genetic diversity estimates in the *Algarvia *population were H = 0.81 ± 0.05, π = 0.003 ± 0.002 and MNPD = 3.03 ± 1.62. Pairwise F_st _values showed that *Algarvia *is significantly differentiated from all breeds except from Garvonesa (F_st _= 0.028, *P *> 0.05).

**Figure 3:**
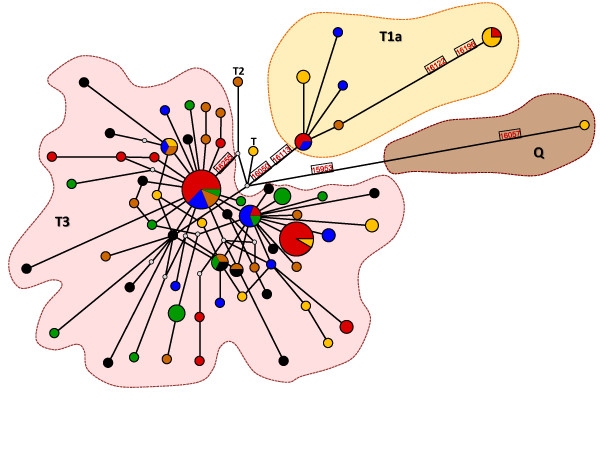
**MJ network of mtDNA haplotypes**. Breeds are colour-coded as follows: Alentejana (orange), *Algarvia *(red), Garvonesa (blue), Mertolenga (brown), Preta (green) and Limousin (black); circle sizes are proportional to haplotype frequencies; major haplogroups are indicated by coloured shadows: European T3 (red), African-derived T1a (orange) and ancestral Mediterranean Q (brown); mutated nt positions that differentiate haplogroups are also shown; theoretical median vectors are represented by grey dots

Haplotype relationships represented in the MJ-network (Figure 3) showed that the most common haplotype in the *Algarvia *population (11 animals, including males AG3M and AG4M) was shared with one Alentejana individual, whereas the second most frequent haplotype (10 animals) was shared with three Garvonesa, two Mertolenga and one Preta individuals. Two *Algarvia *animals (AG40 and AG43) and one Garvonesa shared a T1a haplotype. Interestingly, a T1a haplotype found in one *Algarvia *(AG16) and three Alentejana animals was substantially different from other haplotypes of this haplogroup. This haplotype has a C and an A at positions nt16122 and nt16196, respectively (nt330 and nt404 in Additional file 6 Figure S4), which are characteristic of the African-derived AA mtDNA lineage described in Latin American Creole cattle and also found in Iberia [56]. Although this haplotype lacks the C and T at positions nt16053 and nt16139, respectively (nt261 and nt347 in Additional file 6 Figure S4), that also define AA, it can represent more ancestral Iberian mtDNA lineages [for a discussion see [50]].

### Conservation analysis

Contributions of each breed to the overall genetic diversity are shown in Table 2. Following the Weitzman approach, and based on population pairwise D_A _values, the between-breed influence on diversity (CB) varied from 3.90 (Arouquesa) to 10.23 (Brava de Lide). The within-breed diversity (CW) values varied from -0.62 (Brava de Lide) to 0.43 (Mertolenga). The F_st _value estimated across all breeds and used to calculate the aggregate genetic diversity (D1) was 0.089. Among autochthonous breeds, the lowest value for D1 was found in the Mirandesa breed (0.23) and the highest for the Preta breed (0.92). The influence of the *Algarvia *breed on the overall genetic diversity was intermediate across all estimates except for the allelic diversity-based (c_i_) calculation. *Algarvia *ranked within the six breeds that contributed most to CB (6.18), and showed a lower contribution to both CW (-0.06) and D1 (0.50). When the allelic diversity was considered, *Algarvia *and seven other autochthonous breeds made no contribution to the overall genetic diversity.

**Table 2:** Breed contributions to overall genetic diversity

Population	CB	CW	D1	c_i_, λ = *1*
Alentejana	4.62	-0.17	0.25	0
***Algarvia***	**6.18**	**-0.06**	**0.50**	**0**
Arouquesa	3.90	0.29	0.61	0
Barrosã	4.76	-0.08	0.35	0
Brava de Lide	10.23	-0.62	0.35	0
Garvonesa	8.32	0.18	0.91	22.4
Marinhoa	5.13	-0.09	0.37	0
Maronesa	5.36	0.12	0.59	0
Mertolenga	5.17	0.43	0.85	12.7
Minhota	4.98	0.18	0.61	0
Mirandesa	8.91	-0.61	0.23	12.2
Preta	8.60	0.17	0.92	17.5
Charolais	5.64	-0.11	0.40	5.6
Friesian	9.16	0.16	0.96	24.4
Limousin	5.76	0.20	0.70	5.2

*Algarvia *is represented by the 33 animals identified with STRUCTURE

CB: Weitzman estimate of between-breed genetic diversity; CW: within-breed genetic diversity; D1: aggregate genetic diversity, 0.089*CB + 0.911*CW; ci: contribution of each breed to a pool of maximal genetic diversity [49]

## Discussion

Decisions to initiate costly conservation programmes need to take into account assessment of extant variability and diversity loss, as well as cultural and economic aspects [57-59]. *Algarvia *cattle were adapted to the climatic and geographical conditions of Algarve, a region that is highly susceptible to suffer from climate change [60]. In addition to its cultural and historical relevance, reconstituting this breed could also contribute to reinforce sustainable agriculture, a non negligible component of the economic activity of the region. The use of molecular data to assess the genetic structure of domestic species in conservation programmes has been described [59,61-67]. Analysis of morphological and molecular information provides a more solid basis to define the characteristics of a breed than the use of morphological traits alone. DNA markers were not available at the time when the *Algarvia *breed became nearly extinct, and no biological samples have been preserved. Based on morphology descriptions [22], putative *Algarvia *descendants were identified [27]. As preliminary work towards reconstituting this breed, we used molecular markers to investigate to which extent these animals could be distinguished from other Portuguese cattle. In agreement with their morphological classification [20,23,27,68], genetic distances and factorial correspondence analyses showed a close relationship between *Algarvia *and other breeds of the Aquitanian (Red Convex) group. *Algarvia *and Garvonesa breeds are considered to be descendants of the Alentejana breed [23], and our study shows that *Algarvia *is closely related to these breeds. This genetic proximity probably also reflects recent admixture, because most of the *Algarvia *animals were found in herds predominantly composed of Alentejana animals. Genetic erosion due to crossbreeding is expected to have occurred since the breed began to decline about 35 years ago.

We used assignment tests to determine whether a cluster of putative *Algarvia *animals could be distinguished from the related southern breeds and to identify potential candidates to reconstitute the breed. STRUCTURE results confirmed that the most probable partition of the data agreed with the number of populations tested. Although the *Algarvia *group was the last to emerge as an independent cluster, it was possible to identify 17 cows that belonged to this group with q values > 0.95 without using prior information on populations. With prior information on sample origin, 33 animals (30 cows and three bulls) were classified as *Algarvia*. The partially Bayesian method of GENECLASS resulted in relatively low percentages of animals classified in each breed but, because of the high accuracy, it was useful to confirm reference animals in each population. Even though GENECLASS methods are conservative [55], the results were consistent with those of STRUCTURE with 23 animals assigned to *Algarvia *of which only two (AG34 and AG1M) were not among those selected by STRUCTURE.

Based on their genotypes, three bulls (AG2M, AG3M and AG4M) identified as *Algarvia *can be used to reconstitute the breed. The remaining bull (AG1M) was possibly admixed with Alentejana, according to the STRUCTURE analysis, although with GENECLASS it was assigned to the source population but with a low probability. Overall, a certain amount of convergence of results from independent approaches is noted, since 19 of these animals (16 cows and three bulls) were also identified as belonging to the core group to reconstitute the *Algarvia *breed based on numerical taxonomy analyses of morphological characters [27]. Admixture was detected in several *Algarvia *females, three of which (AG33, AG35 and AG41) did not represent descendants of this breed because they were misclassified. Based on morphology, these animals clustered within a group also containing Alentejana, Garvonesa and Mertolenga animals [27]. The heterogeneous composition of the putative *Algarvia *population is reflected by the lower average genotype membership coefficients when compared to those of breeds having herdbook registries. This result was not surprising given the expected dilution of *Algarvia *through crossbreeding.

The Y-haplotypes of the putative *Algarvia *bulls provided additional evidence of the genetic proximity with Alentejana and Garvonesa breeds, but did not exclude possible admixture with imported breeds such as Limousin or Charolais. mtDNA analysis corroborated the close genetic relationship between the core group of 33 *Algarvia *animals and the Garvonesa breed through their sharing of haplotypes. Common Iberian matrilines (European T3 and African T1a) were found in the *Algarvia *population, as well as a distinct haplotype (AG16) possibly related to the more ancestral African-derived AA haplogroup found in Creole cattle [50]. The use of genotypic data, together with morphological analysis, facilitated the definition of a group of animals that could be used to reconstitute the *Algarvia *breed. How conservative the inclusion criteria should be relative to the acceptable degree of admixture will depend on breeding strategies yet to be defined.

The genetic diversity of the core set of 33 *Algarvia *animals was slightly lower than that found across Portuguese breeds. A significant heterozygote deficit was detected, possibly due to inbreeding, which is consistent with the strong genetic erosion. These results were not unexpected considering the extremely reduced number of extant *Algarvia *descendants. Another possible explanation for HWE deviations could be the sampling of *Algarvia *animals from independent herds, which could generate population subdivision and an increased frequency of homozygotes (e.g. *Wahlund *effect).

Gene diversity-based estimates indicate that the *Algarvia *population makes an intermediate contribution to the overall genetic diversity of Portuguese cattle. In contrast, the allelic diversity-based estimates suggest that the variation found in *Algarvia *is represented in the genetic pool of four other autochthonous breeds. The decision concerning which of these measures should be used in management programs to evaluate breed contributions to overall genetic diversity is not consensual [61]. In the case of Portuguese autochthonous breeds that are considered endangered [4], particularly for the *Algarvia *breed, immediate conservation measures should aim at maximizing gene diversity rather than allelic diversity. This approach would maintain allelic diversity and guarantee a more effective response to selection while controlling inbreeding [61,64].

## Conclusion

Although nearly three decades have elapsed since the *Algarvia *breed was declared effectively extinct, we were able to identify a small group of cows and bulls with phenotypic characteristics of this breed. Analyses of autosomal, maternal and paternal markers have helped refine previous morphological findings to identify 33 animals that can be considered remnants from the *Algarvia *breed. For cultural and economic reasons, reconstituting the *Algarvia *breed is relevant to maintain the distinct regional identity of the Algarve. Molecular analyses have characterized the genetic diversity of the core set of animals which, together with information from conservation analyses, can be used to establish a management program to reconstitute the *Algarvia *breed.

## Competing interests

The authors declare that they have no competing interests.

## Authors' contributions

CG performed assays for Y-chromosome markers and mtDNA sequencing, designed and performed the statistical analyses, drafted and revised the manuscript. MCTP contributed to data analysis, critically reviewed and edited the manuscript. MFS participated in the design of the study and designed and carried out the sampling procedure and selection of the putative *Algarvia *animals. JM and CB contributed the genotype data for *Algarvia*, Garvonesa and Preta animals. DN organised blood preservation and carried out DNA extraction, isolation, purification and analyses. TRF contributed the genotype data for Portuguese autochthonous breeds. AC was the principal investigator who conceived and organized the project, reviewed and edited the manuscript. All authors read and approved the final manuscript.

## Supplementary Material

Additional file 1 **Figure S1 - Neighbour-Joining dendrogram**. The N-J dendogram is based on pairwise D_A _distances among Portuguese cattle, imported breeds and the *Algarvia *population (N = 46); Boostrap values are indicated. Click here for file

Additional file 2 Figure S2 - Results of the factorial correspondence analysis Click here for file

Additional file 3 Table S1 - Estimated genetic diversity for *Algarvia *animals, 11 Portuguese and three imported cattle breeds Click here for file

Additional file 4 **Figure S3 - Distribution of ΔK obtained with STRUCTURE**. Analysis done without prior information on source breeds for K = 1 to K = 9 and calculated as in Evanno *et al*. [42] Click here for file

Additional file 5 Table S2 - GENECLASS analysis of *Algarvia *(N = 46), southern Portuguese breeds (Alentejana, Garvonesa, Mertolenga and Preta) and Limousin cattle Click here for file

Additional file 6 **Figure S4 **- Alignment between *Algarvia *mtDNA *D-loop *sequences and the taurine reference sequence [GenBank: V00654]. The European T3 haplogroup is defined by a C at position nt16255 (nt463 in this figure), whereas the African-derived T1a haplogroup is defined by a T and a C at positions nt16050 and 16113, respectively (nt258 and nt321) Click here for file

## References

[B1] Scherf BD World watch list for domestic animal diversity Book World watch list for domestic animal diversity 2000 3 Rome: Food and Agriculture Organisation of the United Nations

[B2] FAO Measurement of Domestic Animal Diversity - A review of recent diversity studies Book Measurement of Domestic Animal Diversity - A review of recent diversity studies 2004 1 Rome: Food and Agriculture Organisation of the United Nations, Commission on Genetic Resources, Working Group on Animal Genetic Resources 38

[B3] Roosen J Fadlaoui A Bertaglia M Economic evaluation for conservation of farm animal genetic resources J Anim Breed Genet 2005 122 217 228 10.1111/j.1439-0388.2005.00530.x 16060488

[B4] Gama LT Carolino N Costa MS Matos CP Country Report on Farm Animal Genetic Resources Book Country Report on Farm Animal Genetic Resources 2004 Vale de Santarém: Food and Agriculture Organization of the United Nations 68

[B5] Matos CAP Recursos genéticos animais e sistemas de exploração tradicionais em Portugal Arch Zootec 2000 49 363 383

[B6] Fernández A Viana JL Iglesias A Sanchez L Genetic variability and phylogenetic relationships between ten native cattle breeds from Galicia and the north of Portugal Arch Zootec 1998 47 61 71

[B7] Beja-Pereira A Alexandrino P Bessa I Carretero Y Dunner S Ferrand N Jordana J Laloe D Moazami-Goudarzi K Sanchez A Canon J Genetic characterization of southwestern European bovine breeds: a historical and biogeographical reassessment with a set of 16 microsatellites J Hered 2003 94 243 250 10.1093/jhered/esg055 12816965

[B8] Cañon J Alexandrino P Bessa I Carleos C Carretero Y Dunner S Ferrand N Garcia D Jordana J Laloë D Pereira A Sanchez A Moazami-Goudarzi K Genetic diversity measures of local European beef cattle breeds for conservation purposes Genet Sel Evol 2001 33 311 332 10.1186/1297-9686-33-3-311 11403750 PMC2705410

[B9] Cymbron T Freeman AR Isabel Malheiro M Vigne JD Bradley DG Microsatellite diversity suggests different histories for Mediterranean and Northern European cattle populations Proc Biol Sci 2005 272 1837 1843 10.1098/rspb.2005.3138 16096097 PMC1559860

[B10] Ginja C Identificação de raças bovinas Portuguesas atravès da utilização de marcadores moleculares Universidade de Trás-os-Montes e Alto Douro 2002

[B11] Mateus JC Eding H Penedo MC Rangel-Figueiredo MT Contributions of Portuguese cattle breeds to genetic diversity using marker-estimated kinships Anim Genet 2004 35 305 313 10.1111/j.1365-2052.2004.01168.x 15265070

[B12] Mateus JC Penedo MC Alves VC Ramos M Rangel-Figueiredo T Genetic diversity and differentiation in Portuguese cattle breeds using microsatellites Anim Genet 2004 35 106 113 10.1111/j.1365-2052.2004.01089.x 15025569

[B13] Cymbron T Loftus RT Malheiro MI Bradley DG Mitochondrial sequence variation suggests an African influence in Portuguese cattle Proc R Soc Lond B Biol Sci 1999 266 597 603 10.1098/rspb.1999.0678 PMC1689806 10212450

[B14] Ginja C Penedo MC Melucci L Quiroz J Martinez Lopez OR Revidatti MA Martinez-Martinez A Delgado JV Gama LT Origins and genetic diversity of New World Creole cattle: inferences from mitochondrial and Y chromosome polymorphisms Anim Genet 2010 41 128 141 10.1111/j.1365-2052.2009.01976.x 19817725

[B15] Ginja C Gama LT Penedo MCT Y chromosome haplotype analysis in Portuguese cattle breeds using SNPs and STRs J Hered 2009 100 148 157 10.1093/jhered/esn080 18832111

[B16] Achilli A Olivieri A Pellecchia M Uboldi C Colli L Al-Zahery N Accetturo M Pala M Kashani BH Perego UA Battaglia V Fornarino S Kalamati J Houshmand M Negrini R Semino O Richards M Macaulay V Ferretti L Bandelt H-J Ajmone-Marsan P Torroni A Mitochondrial genomes of extinct aurochs survive in domestic cattle Curr Biol 2008 18 157 158 10.1016/j.cub.2008.01.019 18302915

[B17] Baganha DA Resposta ao Questionário dirigido aos proprietários rurais e lavradores de todos os concelhos do reino Book Resposta ao Questionário dirigido aos proprietários rurais e lavradores de todos os concelhos do reino 1868

[B18] Cordeiro JP As raças bovinas em Portugal. Sua origem OMédico Veterinário 1986 29 39

[B19] Lima BS Os escritos de Silvestre Bernardo Lima. Bovídeos e Suídeos Boletim do Ministério da Agricultura 1919 I 1 266

[B20] Lima S Recenseamento Geral de Gados no Continente do Reino de Portugal em 1870 Lisboa: Imprensa Nacional 1873 1

[B21] Nogueira JVP Les animaux agricoles Le Portugal au Point de Vue Agricole 1900 Lisbonne: Imprimerie Nationale 310

[B22] Pereira MT A raça bovina Algarvia Boletim Pecuário da Direcção Geral dos Serviços Pecuários 1960 1 137 189

[B23] Vale JM Bovinos Gado Bissulco:suinos bovinos, arietinos caprinos 1949 Lisboa: Livraria Sá da Costa 81 168

[B24] Felius M Genus Bos: Cattle breeds of the world New Jersey: Merck & Co. Inc 1985 1

[B25] Rosado J Almeida JM Gouveia JM Sobral M Lobo JMS Rodrigues A Raça bovina Algarvia. Inquérito para a sua caracterização e seu sistema de exploração Bovinos em Portugal 1981 Lisboa: Direcção Geral dos Serviços Veterinários 217 222

[B26] Sobral MF Cravador A Navas D Roberto C C R Lima MB Classificação e caracterização morfológica de raças bovinas autóctones nacionais utilizando a taxonomia numérica Rev Port Zootec 2001 2 123 137

[B27] Sobral MF Roberto C Navas D Palmilha I Lima MB Cravador A Identification of descendants of an extinct bovine population from the Algarve region of Portugal using numerical taxonomy analysis of morphological traits J Anim Breed Genet 2009 126 319 326 10.1111/j.1439-0388.2008.00787.x 19630883

[B28] Hughes SS STRand Nucleic Acids Analysis Software 2000 1.2.9011052354

[B29] Mateus JC Eding H Penedo MC Rangel-Figueiredo MT Contributions of Portuguese cattle breeds to genetic diversity using marker-estimated kinships Anim Genet 2004 35 305 313 10.1111/j.1365-2052.2004.01168.x 15265070

[B30] Peakall R Smouse PE Genalex 6: genetic analysis in Excel. Population genetic software for teaching and research Mol Ecol Notes 2006 6 288 295 10.1111/j.1471-8286.2005.01155.x PMC3463245 22820204

[B31] Raymond M Rousset F GENEPOP: population genetics software for exact tests and ecumenicism J Hered 1995 86 248 249

[B32] Belkhir K Borsa P Chikhi L Raufaste N Bonhomme F GENETIX: logiciel sous Windows TM pour la génétique des populations 4.02 Laboratoire Genome Populations,Interactions CNRS, Université de Montpellier II 1996 2004

[B33] Nei M Analysis of gene diversity in subdivided populations Proc Natl Acad Sci USA 1973 70 3321 3323 10.1073/pnas.70.12.3321 4519626 PMC427228

[B34] Weir BS Cockerham CC Estimating F-statistics for the analysis of population structure Evolution 1984 38 1358 1370 10.2307/2408641 28563791

[B35] Goudet J FSTAT a program to estimate and test gene diversities and fixation indices Updated from Goudet 1995 2001 2.9.3

[B36] Saitou N Nei M The neighbor-joining method: a new method for reconstructing phylogenetic trees Mol Biol Evol 1987 4 406 425 3447015 10.1093/oxfordjournals.molbev.a040454

[B37] Nei M Tajima F Tateno Y Accuracy of estimated phylogenetic trees from molecular data J Mol Evol 1983 19 153 170 10.1007/BF02300753 6571220

[B38] Langella O POPULATIONS 1.2.28 CNRS UPR9034 1999

[B39] Page RD TREEVIEW: an application to display phylogenetic trees on personal computers Comput Appl Biosci 1996 12 357 358 8902363 10.1093/bioinformatics/12.4.357

[B40] Pritchard JK Wen W STRUCTURE software. Users Manual 2004 2

[B41] Falush D Stephens M Pritchard JK Inference of population structure using multilocus genotype data: linked loci and correlated allele frequencies Genetics 2003 164 1567 1587 12930761 10.1093/genetics/164.4.1567 PMC1462648

[B42] Evanno G Regnaut S Goudet J Detecting the number of clusters of individuals using the software STRUCTURE: a simulation study Mol Ecol 2005 14 2611 2620 10.1111/j.1365-294X.2005.02553.x 15969739

[B43] Piry S Alapetite A Cornuet JM Paetkau D Baudouin L Estoup A GENECLASS2: a software for genetic assignment and first-generation migrant detection J Hered 2004 95 536 539 10.1093/jhered/esh074 15475402

[B44] Rannala B Mountain JL Detecting immigration by using multilocus genotypes Proc Natl Acad Sci USA 1997 94 9197 9201 10.1073/pnas.94.17.9197 9256459 PMC23111

[B45] Paetkau D Slade R Burden M Estoup A Genetic assignment methods for the direct real-time estimation of migration rate: a simulation-based exploration of accuracy and power Mol Ecol 2004 13 55 65 10.1046/j.1365-294X.2004.02008.x 14653788

[B46] Cornuet JM Piry S Luikart G Estoup A Solignac M New methods employing multilocus genotypes to select or exclude populations as origins of individuals Genetics 1999 153 1989 2000 10581301 10.1093/genetics/153.4.1989 PMC1460843

[B47] Ollivier L Foulley JL Aggregate diversity: new approach combining within- and between-breed genetic diversity Livest Prod Sci 2005 95 247 254 10.1016/j.livprodsci.2005.01.005

[B48] Perez-Figueroa A Saura M Fernández J Toro MA Caballero A METAPOP - A software for the management and analysis of subdivided populations in conservation programs Conserv Genet 2009 10 1097 1099 10.1007/s10592-008-9718-7

[B49] Caballero A Toro MA Analysis of genetic diversity for the management of conserved subdivided populations Conserv Genet 2002 3 289 299 10.1023/A:1019956205473

[B50] Ginja C Penedo MC Melucci L Quiroz J Matrinez Lopez OR Revidatti MA Martinez AM Delgado JV Gama L Origins and genetic diversity of New World Creole cattle: inferences from mitochondrial and Y chromosome polymorphisms Anim Genet 2010 41 128 141 10.1111/j.1365-2052.2009.01976.x 19817725

[B51] Anderson S de Bruijn MH Coulson AR Eperon IC Sanger F Young IG Complete sequence of bovine mitochondrial DNA. Conserved features of the mammalian mitochondrial genome J Mol Biol 1982 156 683 717 10.1016/0022-2836(82)90137-1 7120390

[B52] Schneider S Roessli D Excoffier L Arlequin ver. 2.000: A software for population genetics data analysis 2000 Genetics and Biometry Laboratory, University of Geneva

[B53] Tamura K Nei M Estimation of the number of nucleotide substitutions in the control region of mitochondrial DNA in humans and chimpanzees Mol Biol Evol 1993 10 512 526 8336541 10.1093/oxfordjournals.molbev.a040023

[B54] Bandelt HJ Forster P Rohl A Median-joining networks for inferring intraspecific phylogenies Mol Biol Evol 1999 16 37 48 10331250 10.1093/oxfordjournals.molbev.a026036

[B55] Manel S Berthier P Luikart G Detecting wildlife poaching: Identifying the origin of individuals with Bayesian Assignment tests and multilocus genotypes Conserv Biol 2002 16 650 659 10.1046/j.1523-1739.2002.00576.x

[B56] Miretti MM Dunner S Naves M Contel EP Ferro JA Predominant African-derived mtDNA in Caribbean and Brazilian Creole cattle is also found in Spanish cattle (*Bos taurus*) J Hered 2004 95 450 453 10.1093/jhered/esh070 15388773

[B57] Bruford MW Simm G, Villanueva B, Townsend S Conservation genetics of UK livestock: from molecules to management Farm animal genetic resources 2004 Nottingham: Nottingham University Press 151 169

[B58] Tapio I Varv S Bennewitz J Maleviciute J Fimland E Grislis Z Meuwissen TH Miceikiene I Olsaker I Viinalass H Vilkki J Kantanen J Prioritization for conservation of northern European cattle breeds based on analysis of microsatellite data Conserv Biol 2006 20 1768 1779 10.1111/j.1523-1739.2006.00488.x 17181812

[B59] Bennewitz J Meuwissen TH A novel method for the estimation of the relative importance of breeds in order to conserve the total genetic variance Genet Sel Evol 2005 37 315 337 10.1186/1297-9686-37-4-315 15823238 PMC2697237

[B60] Diffenbaugh NS Pal JS Giorgi F Gao X Heat stress intensification in the Mediterranean climate change hotspot Geophys Res Lett 2007 34 doi:10.1029/2007GL030000 10.1029/2007GL030000

[B61] Caballero A Rodriguez-Ramilo ST Avila V Fernandez J Management of genetic diversity of subdivided populations in conservation programmes Conserv Genet 2010 11 409 419 10.1007/s10592-009-0020-0

[B62] Chikhi L Goossens B Treanor A Bruford MW Population genetic structure of and inbreeding in an insular cattle breed the Jersey and its implications for genetic resource management Heredity 2004 92 396 401 10.1038/sj.hdy.6800433 15014423

[B63] Tapio I Varv S Bennewitz J Maleviciute J Fimland E Grislis Z Meuwissen TH Miceikiene I Olsaker I Viinalass H Vilkki J Kantanen J Prioritization for conservation of northern European cattle breeds based on analysis of microsatellite data Conserv Biol 2006 20 1768 1779 10.1111/j.1523-1739.2006.00488.x 17181812

[B64] Toro MA Caballero A Characterization and conservation of genetic diversity in subdivided populations Philos Trans R Soc Lond B Biol Sci 2005 360 1367 1378 10.1098/rstb.2005.1680 16048780 PMC1569508

[B65] Bray TC Chikhi L Sheppy AJ Bruford MW The population genetic effects of ancestry and admixture in a subdivided cattle breed Anim Genet 2009 40 393 400 10.1111/j.1365-2052.2009.01850.x 19397525

[B66] Fabuel E Barragan C Silio L Rodriguez MC Toro MA Analysis of genetic diversity and conservation priorities in Iberian pigs based on microsatellite markers Heredity 2004 93 104 113 10.1038/sj.hdy.6800488 15150539

[B67] Berthouly C Rognon X Nhu Van T Berthouly A Thanh Hoang H Bed'Hom B Laloe D Vu Chi C Verrier E Maillard JC Genetic and morphometric characterization of a local Vietnamese swamp buffalo population J Anim Breed Genet 127 74 84 10.1111/j.1439-0388.2009.00806.x 20074189

[B68] Tierno J Notas sobre Portugal - Industria Pecuária Lisboa: Imprensa Nacional 1908 1

