# Supplementary figures and images for: Molecular genetic analysis of a cattle population to reconstitute the extinct Algarvia breed
Source: Genet Sel Evol. 2010 Jun 11;42(1):18. doi: 10.1186/1297-9686-42-18 (PMC2903498; doi:10.1186/1297-9686-42-18)

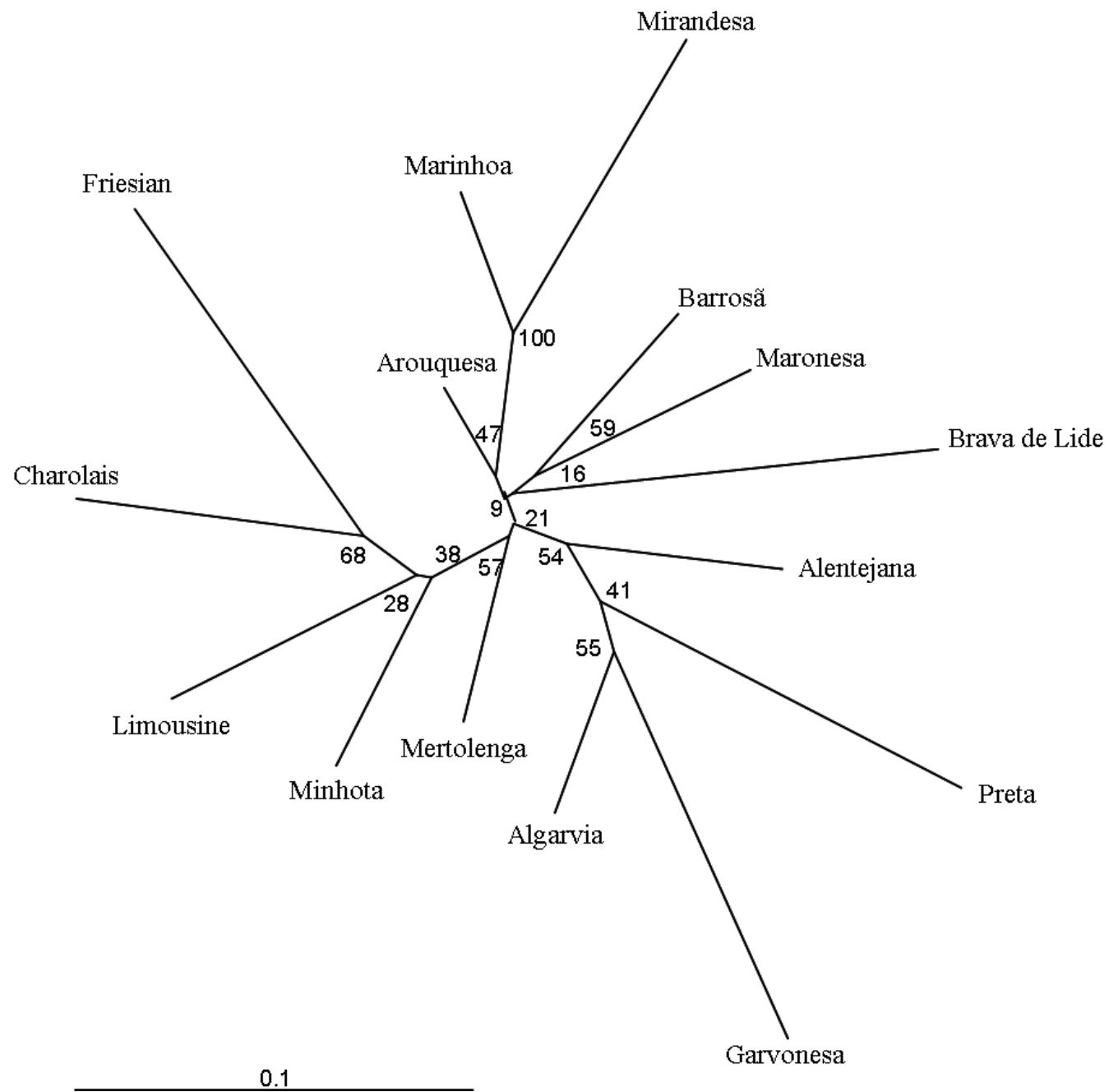

Supplement: Additional file 1 — Figure S1 - Neighbour-Joining dendrogram. The N-J dendogram is based on pairwise DA distances among Portuguese cattle, imported breeds and the Algarvia population (N = 46); Boostrap values are indicated. [file 1297-9686-42-18-S1.PDF]

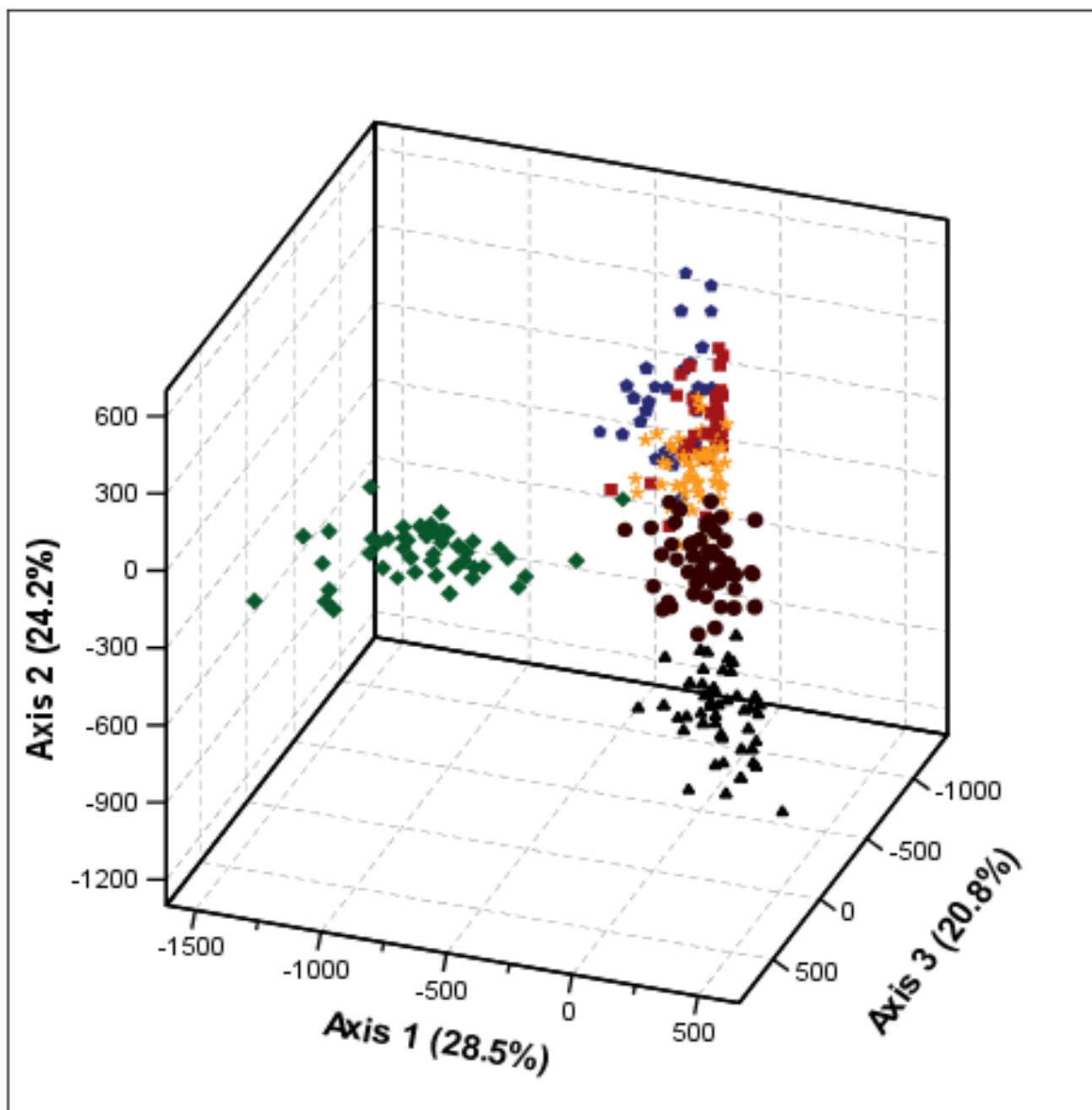

- Legend
- ★ Alentejana
  - Algarvia
  - ⬠ Garvonesa
  - ▲ Limousin
  - Mertolenga
  - ◆ Preta

Supplement: Additional file 2 — Figure S2 - Results of the factorial correspondence analysis [file 1297-9686-42-18-S2.PDF]

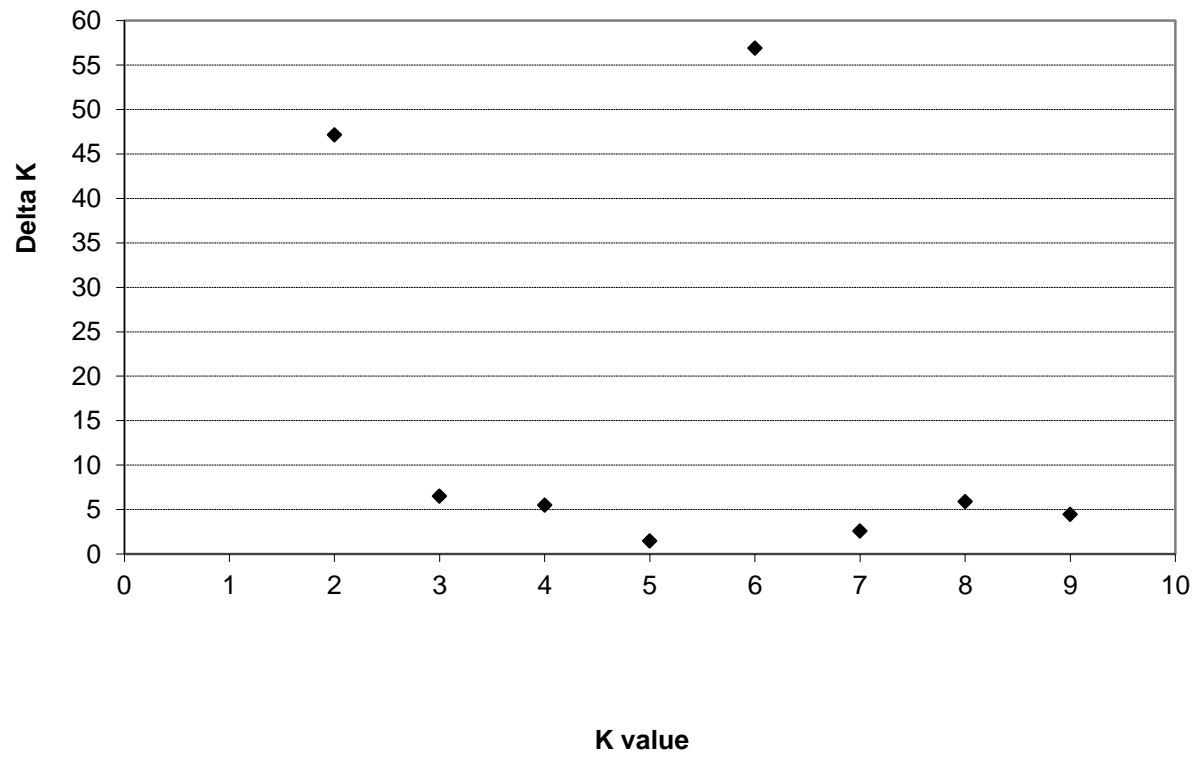

Supplement: Additional file 4 — Figure S3 - Distribution of ΔK obtained with STRUCTURE. Analysis done without prior information on source breeds for K = 1 to K = 9 and calculated as in Evanno et al. [42] [file 1297-9686-42-18-S4.PDF]
