# Supplementary material for: Molecular genetic analysis of a cattle population to reconstitute the extinct Algarvia breed
Source: Genet Sel Evol. 2010 Jun 11;42(1):18. doi: 10.1186/1297-9686-42-18 (PMC2903498; doi:10.1186/1297-9686-42-18)
Supplement: Additional file 3 — Table S1 - Estimated genetic diversity for Algarvia animals, 11 Portuguese and three imported cattle breeds [file 1297-9686-42-18-S3.PDF]

**Population**	**N**	**MNA**	SD	**PA**	**R_t**	SD	**H_O**	SD	**H_e**	SD	**f_m**	**F_{is}**	**HWEd**	**LD**
**Autochthonous**														
Alentejana	50	6.7	2.1	1	5.8	1.5	0.642	0.123	0.680	0.121	0.327	0.056^{**}	7	1
Algarvia	*33*[a]	*6.0*	*1.6*	*10*	*5.7*	*1.4*	*0.635*	*0.188*	*0.691*	*0.102*	*0.322*	*0.083****	*10*	
Arouquesa	50	7.2	2.6	1	6.4	2.1	0.716	0.131	0.725	0.111	0.282	0.012	5	
Barrosã	50	6.7	1.9	2	5.9	1.5	0.666	0.150	0.689	0.144	0.318	0.034^{*}	3	
Brava de Lide	40	5.8	1.9		5.3	1.6	0.544	0.170	0.637	0.167	0.371	0.148^{***}	15	4
Garvonesa	29	6.5	2.2	5	6.2	2.0	0.680	0.177	0.715	0.136	0.296	0.050^{*}	6	
Marinhoa	51	6.7	1.9	1	5.8	1.3	0.667	0.111	0.688	0.097	0.319	0.031^{*}	6	
Maronesa	47	6.5	2.2	1	5.8	1.9	0.725	0.120	0.709	0.100	0.299	-0.023	0	
Mertolenga	50	7.7	2.5	2	6.8	2.0	0.684	0.102	0.739	0.107	0.269	0.074^{***}	9	
Minhota	50	7.5	2.3	1	6.6	1.8	0.690	0.134	0.715	0.110	0.293	0.035^{**}	2	
Mirandesa	50	5.7	1.3		4.9	0.9	0.621	0.114	0.637	0.099	0.369	0.026	1	2
Preta	47	7.1	2.8	7	6.3	2.2	0.636	0.132	0.714	0.087	0.294	0.110^{***}	13	4
**Exotic**														
Charolais	45	6.7	2.2	1	6.0	1.8	0.677	0.171	0.686	0.149	0.321	0.014	3	1
Friesian	35	7.0	2.3	4	6.4	1.8	0.702	0.141	0.712	0.101	0.298	0.015	4	
Limousin	48	7.0	2.7	2	6.2	1.9	0.709	0.089	0.717	0.081	0.291	0.011	2	
**Overall**[b]	**642**	**6.8**	**0.6**	**28**	**6.0**	**1.8**	**0.668**	**0.047**	**0.697**	**0.030**	**0.310**	**0.042**	**76**	**12**

[a]Animals identified as belonging to the core group to recover *Algarvia*; [b]Overall values across breeds without *Algarvia*; *($P < 0.05$); **($P < 0.01$); ***($P < 0.001$); N: sample size; MNA: mean number of alleles; PA: number of population-specific alleles; R_t: allelic richness; H_o: observed heterozygosity; H_e: unbiased expected heterozygosity; f_m: molecular coancestry; F_{is}: inbreeding coefficient; HWEd: number of loci showing significant ($P < 0.05$) HWE deviation due to heterozygote deficit; LD: number of loci pairs showing significant ($P < 0.001$) linkage disequilibrium; SD: standard deviation
