# Supplementary material for: Molecular genetic analysis of a cattle population to reconstitute the extinct Algarvia breed
Source: Genet Sel Evol. 2010 Jun 11;42(1):18. doi: 10.1186/1297-9686-42-18 (PMC2903498; doi:10.1186/1297-9686-42-18)
Supplement: Additional file 5 — Table S2 - GENECLASS analysis of Algarvia (N = 46), southern Portuguese breeds (Alentejana, Garvonesa, Mertolenga and Preta) and Limousin cattle [file 1297-9686-42-18-S5.PDF]

	Maximum Likelihood	Threshold	Partially Bayesian method (Paetkau *et al.* 2004)			
			Correct	Misassigned	Excluded	Multiple
Algarvia	89.1					
		$P < 0.05$	50.0	0.0	19.6	30.4
		$P < 0.01$	39.1	2.2	4.3	54.3
		$P < 0.001$	21.7	0.0	2.2	76.1
**Overall**	95.9					
		$P < 0.05$	61.1	1.1	16.3	21.5
		$P < 0.01$	59.6	0.4	4.8	35.2
		$P < 0.001$	34.8	0.0	0.7	64.4

Proportions of individuals correctly classified in source populations, misassigned, excluded from all source populations and classified in several populations (multiple) for thresholds of 0.05, 0.01 and 0.001 are shown.
