# Supplementary material for: Molecular genetic analysis of a cattle population to reconstitute the extinct Algarvia breed
Source: Genet Sel Evol. 2010 Jun 11;42(1):18. doi: 10.1186/1297-9686-42-18 (PMC2903498; doi:10.1186/1297-9686-42-18)
Supplement: Additional file 6 — Figure S4 - Alignment between Algarvia mtDNA D-loop sequences and the taurine reference sequence [GenBank: V00654]. The European T3 haplogroup is defined by a C at position nt16255 (nt463 in this figure), whereas the African-derived T1a haplogroup is defined by a T and a C at positions nt16050 and 16113, respectively (nt258 and nt321) [file 1297-9686-42-18-S6.PDF]

```
                10         20         30         40         50
        ....|....| ....|....| ....|....| ....|....| ....|....|
V00654  ACACTATTAA TATAGTTCCA TAAATACAAA GAGCCTTATC AGTATTAAAT
AG01    .......... .......... .......... .......... ..........
AG02    .......... .......... .......... .......... ..........
AG04    .......... .......... .......... .......... ..........
AG05    .......... .......... .......... .......... ..........
AG06    .......... .......... .......... .......... ..........
AG07    .......... .......... .......... .......... ..........
AG08    .......... .......... .......... .......... ..........
AG09    .......... .......... .......... .......... ..........
AG10    .......... .......... .......... .......... ..........
AG11    .......... .......... .......... .......... ..........
AG13    .......... .......... .......... .......... ..........
AG14    .......... .......... .......... .......... ..........
AG15    .......... .......... .......... .......... ..........
AG16    .......... .......... .......... .......... ..........
AG18    .......... .......... .......... .......... ..........
AG20    .......... .......... .......... .......... ..........
AG21    .......... .......... .......... .......... ..........
AG22    .......... .......... .......... .......... ..........
AG24    .......... .......... .......... .......... ..........
AG25    .......... .......... .......... .......... ..........
AG26    .......... .......... .......... .......... ..........
AG27    .......... .......... .......... .......... ..........
AG28    .......... .......... .......... .......... ..........
AG29    .......... .......... .......... .......... ..........
AG30    .......... .......... .......... .......... ..........
AG31    .......... .......... .......... .......... ..........
AG40    .......... .......... .......... .......... ..........
AG42    .......... .......... .......... .......... ..........
AG43    .......... .......... .......... .......... ..........
AG1M    .......... .......... .......... .......... ..........
AG2M    .......... .......... .......... .......... ..........
AG3M    .......... .......... .......... .......... ..........
AG4M    .......... .......... .......... .......... ..........

                60         70         80         90        100
        ....|....| ....|....| ....|....| ....|....| ....|....|
V00654  TTATCAAAAA TCCCAATAAC TCAACACAGA ATTTGCACCC TAACCAAATA
AG01    .......... .......... .......... .......... ..........
AG02    .......... .......... .......... .......... ..........
AG04    .......... .......... .......... .......... ..........
AG05    .......... .......... .......... .......... ..........
AG06    .......... .......... .......... .......... ..........
AG07    .......... .......... .......... .......... ..........
AG08    .......... .......... .......... .......... ..........
AG09    .......... .......... .......... .......... ..........
AG10    .......... .......... .......... .......... ..........
AG11    .......... .......... .......... .......... ..........
AG13    .......... .......... .......... .......... ..........
AG14    .......... .......... .......... .......... ..........
AG15    .......... .......... .......... .......... ..........
AG16    .......... .......... .......... .......... ..........
AG18    .......... .......... .......... .......... ..........
AG20    .......... .......... .......... .......... ..........
AG21    .......... .......... .......... .......... ..........
AG22    .......... .......... .......... .......... ..........
AG24    .......... .......... .......... .......... ..........
AG25    .......... .......... .......... .......... ..........
AG26    .......... .......... .......... .......... ..........
AG27    .......... .......... .......... .......... ..........
AG28    .......... .......... .......... .......... ..........
AG29    .......... .......... .......... .......... ..........
AG30    .......... .......... .......... .......... ..........
AG31    .......... .......... .......... .......... ..........
AG40    .......... .......... .......... .......... ..........
AG42    .......... .......... .......... .......... ..........
AG43    .......... .......... .......... .......... ..........
AG1M    .......... .......... .......... .......... ..........
AG2M    .......... .......... .......... .......... ..........
AG3M    .......... .......... .......... .......... ..........
AG4M    .......... .......... .......... .......... ..........
```

```
                     110        120        130        140        150
          ....|....| ....|....| ....|....| ....|....| ....|....|
V00654    TTACAAACAC CACTAGCTAA CATAACACGC CCATACACAG ACCACAGAAT
AG01      .......... .......... .......... .......... ..........
AG02      .......... .......... .......... .......... ..........
AG04      .......... .......... .......... .......... ..........
AG05      .......... .......... .......... .......... ..........
AG06      .......... .......... .......... .......... ..........
AG07      .......... .......... .......... .......... ..........
AG08      .......... .......... .......... .......... ..........
AG09      .......... .......... .......... .......... ..........
AG10      .......... .......... .......... .......... ..........
AG11      .......... .......... .......... .......... ..........
AG13      .......... .......... .......... .......... ..........
AG14      .......... .......... .......... .......... ..........
AG15      .......... .......... .......... .......... ..........
AG16      .......... .......... .......... .......... ..........
AG18      .......... .......... .......... .......... ..........
AG20      .......... .......... .......... .......... ..........
AG21      .......... .......... .......... .......... ..........
AG22      .......... .......... .......... .......... ..........
AG24      .......... .......... .......... .......... ..........
AG25      .......... .......... .......... .......... ..........
AG26      .......... .......... .......... .......... ..........
AG27      .......... .......... .......... .......... ..........
AG28      .......... .......... .......... .......... ..........
AG29      .......... .......... .......... .......... ..........
AG30      .......... .......... .......... .......... ..........
AG31      .......... .......... .......... .......... ..........
AG40      .......... .......... .......... .......... ..........
AG42      .......... .......... .......... .......... ..........
AG43      .......... .......... .......... .......... ..........
AG1M      .......... .......... .......... .......... ..........
AG2M      .......... .......... .......... .......... ..........
AG3M      .......... .......... .......... .......... ..........
AG4M      .......... .......... .......... .......... ..........

                     160        170        180        190        200
          ....|....| ....|....| ....|....| ....|....| ....|....|
V00654    GAATTACCTA CGCAAGGGGT AATGTACATA ACATTAATGT AATAAAGACA
AG01      .......... .......... .......... .......... ..........
AG02      .......... .......... .......... .......... ..........
AG04      .......... .......... .......... .......... ..........
AG05      .......... .......... .......... .......... ..........
AG06      .......... .......... .......... .......... ..........
AG07      .......... .......... .......... .......... ..........
AG08      .......... .......... .......... .......... ..........
AG09      .......... .......... .......... .......... ..........
AG10      .......... .......... .......... .......... ..........
AG11      .......... .......... .......... .......... ..........
AG13      .......... .......... .......... .......... ..........
AG14      .......... .......... .......... .......... ..........
AG15      .......... .......... .......... .......... ..........
AG16      .......... .......... .G........ .......... ..........
AG18      .......... .......... .......... .......... ..........
AG20      .......... .......... .......... .......... ..........
AG21      .......... .......... .......... .......... ..........
AG22      .......... .......... .......... .......... ..........
AG24      .......... .......... .......... .......... ..........
AG25      .......... .......... .......... .......... ..........
AG26      .......... .......... .......... .......... ..........
AG27      .......... .......... .......... .......... ..........
AG28      .......... .......... .......... .......... ..........
AG29      .......... .......... .......... .......... ..........
AG30      .......... .......... .......... .......... ..........
AG31      .......... .......... .......... .......... ..........
AG40      .......... .......... .......... .......... ..........
AG42      .......... .......... .......... .......... ..........
AG43      .......... .......... .......... .......... ..........
AG1M      .......... .......... .......... .......... ..........
AG2M      .......... .......... .......... .......... ..........
AG3M      .......... .......... .......... .......... ..........
AG4M      .......... .......... .......... .......... ..........
```

```
                210        220        230        240        250
       ....|....| ....|....| ....|....| ....|....| ....|....|
V00654 TAATATGTAT ATAGTACATT AAATTATATG CCCCATGCAT ATAAGCAAGT
AG01   .......... .......... .......... .......... ..........
AG02   .......... .......... .......... .......... ..........
AG04   .......... .......... .......... .......... ..........
AG05   .......... .......... .......... .......... ..........
AG06   .......... .......... .......... .......... ..........
AG07   .......... .......... .......... .......... ..........
AG08   .......... .......... .......... .......... ..........
AG09   .......... .......... .......... .......... ..........
AG10   .......... .......... .......... .......... ..........
AG11   .......... .......... .......... .......... ..........
AG13   .......... .......... .......... .......... ..........
AG14   .......... .......... .......... .......... ..........
AG15   .......... .......... .......... .......... ..........
AG16   .......... .......... .......... .......... ..........
AG18   .......... .......... .......... .......... ..........
AG20   .......... .......... .......... .......... ..........
AG21   .......... .......... .......... .......... ..........
AG22   .......... .......... .......... .......... ..........
AG24   .......... .......... .......... .......... ..........
AG25   .......... .......... .......... .......... ..........
AG26   .......... .......... .......... .......... ..........
AG27   .......... .......... .........A .......... ..........
AG28   .......... .......... .........A .......... ..........
AG29   .......... .......... .......... .......... ..........
AG30   .......... .......... .......... .......... ..........
AG31   .......... .......... .......... .......... ..........
AG40   .......... .......... .......... .......... ..........
AG42   .......... .......... .......... .......... ..........
AG43   .......... .......... .......... .......... ..........
AG1M   .......... .......... .......... .......... ..........
AG2M   .......... .......... .......... .......... ..........
AG3M   .......... .......... .......... .......... ..........
AG4M   .......... .......... .......... .......... ..........
```

```
                260        270        280        290        300
       ....|....| ....|....| ....|....| ....|....| ....|....|
V00654 ACATGACCTC TATAGCAGTA CATAATACAT ATAATTATTG ACTGTACATA
AG01   .......... .......... .......... .......... ..........
AG02   .......... .......... .......... .......... ..........
AG04   .......... .......... .......... .......... ..........
AG05   .......... .......... .......... .......... ..........
AG06   .......... .......... .......... .......... ..........
AG07   .......... .......... .......... .......... ..........
AG08   .......... .......... .......... .......... ..........
AG09   .......... .......... .......... .......... ..........
AG10   .......... .......... .......... .......... ..........
AG11   .......... .......... .......... .......... ..........
AG13   .......... .......... .......... .......... ..........
AG14   .......... .......... .......... .......... ..........
AG15   .......... .......... .......... .......... ..........
AG16   .......T.. .......... .......... ......G... ..........
AG18   .......... .......... .......... .......... ..........
AG20   .......... .......... .......... .......... ..........
AG21   .......... .......... .......... .......... ..........
AG22   .......... .......... .......... .......... ..........
AG24   .......... .......... .......... .......... ..........
AG25   .......... .......... .......... .......... ..........
AG26   .......... .......... .......... .......... ..........
AG27   .......... .......... .......... .......... ..........
AG28   .......... .......... .......... .......... ..........
AG29   .......... .......... .......... .........A ..........
AG30   .......... .......... .......... .......... ..........
AG31   .......... .......... .......... .......... ..........
AG40   .......T.. .......... .......... .......... ..........
AG42   .......... .......... .......... .......... ..........
AG43   .......T.. .......... .......... .......... ..........
AG1M   .......... .......... .......... .......... ..........
AG2M   .......... .......... .......... .......... ..........
AG3M   .......... .......... .......... .......... ..........
AG4M   .......... .......... .......... .......... ..........
```

```
                                  310         320         330         340         350
         .....|....| .....|....| .....|....| .....|....| .....|....|
V00654   GTACATTATG TCAAATTCAT TCTTGATAGT ATATCTATTA TATATTCCTT
AG01     .......... .......... .......... .......... ..........
AG02     .......... .......... .......... .......... ..........
AG04     .......... .......... .......... .......... ..........
AG05     .......... .......... .......... .......... ..........
AG06     .......... .......... .......... .......... ..........
AG07     .......... .......... .......... .......... ..........
AG08     .......... .......... .......... .......... ..........
AG09     .......... .......... .......... .......... ..........
AG10     .......... .......... .......... .......... ..........
AG11     .......... .......... .......... .......... ..........
AG13     .......... .......... .......... .......... ..........
AG14     .......... .......... .......... .......... ..........
AG15     .......... .......... .......... .......... ..........
AG16     .......... .......... C........C ....T..... ..........
AG18     .......... .......... .......... .......... ....C.....
AG20     .......... .......... .......... .......... ..........
AG21     .......... .......... .......... .......... ..........
AG22     .......... .......... .......... .......... ..........
AG24     .......... .......... .......... .......... ..........
AG25     .......... .......... .......... .......... ..........
AG26     .......... .......... .......... .......... ..........
AG27     .......... .......... C......... .......... ..........
AG28     .......... .......... C......... .......... ..........
AG29     .......... .......... .......... .......... ....C.....
AG30     .......... .......... .......... .......... ..........
AG31     .......... .......... .......... .......... ..........
AG40     .......... .......... C......... .......... ..........
AG42     .......... .......... .......... .......... ..........
AG43     .......... .......... C......... .......... ..........
AG1M     .......... .......... .......... .......... ..........
AG2M     .......... .......... .......... .......... ..........
AG3M     .......... .......... .......... .......... ..........
AG4M     .......... .......... .......... .......... ..........

                                  360         370         380         390         400
         .....|....| .....|....| .....|....| .....|....| .....|....|
V00654   ACCATTAGAT CACGAGCTTA ATTACCATGC CGCGTGAAAC CAGCAACCCG
AG01     .......... .......... .......... .......... ..........
AG02     .......... .......... .......... .......... ..........
AG04     .......... .......... .......... .......... ..........
AG05     .......... .......... .......... .......... ..........
AG06     .......... .......... .......... .......... ..........
AG07     .......... .......... .......... .......... ..........
AG08     .......... .......... ..C....... .......... ..........
AG09     .......... .......... ..C....... .......... ..........
AG10     .......... .......... .......... .......... ..........
AG11     .......... .......... .......... .......... ..........
AG13     .......... .......... ..C....... .......... ..........
AG14     .......... .......... ..C....... .......... ..........
AG15     .......... .......... .......... .......... ..........
AG16     .......... .......... .......... .......... ..........
AG18     .......... .......... .......... .......... ..........
AG20     .......... .......... ..C....... .......... ..........
AG21     .......... .......... .......... .......... ..........
AG22     .......... .......... ..C....... .......... ..........
AG24     .......... .......... ..C....... .......... ..........
AG25     .......... .......... .......... .......... ..........
AG26     .......... .......... ..C....... .......... ..........
AG27     .......... .......... .......... .......... ..........
AG28     .......... .......... .......... .......... ..........
AG29     .......... .......... .......... .......... ..........
AG30     .......... .......... .......... .......... ..........
AG31     .......... .......... ..C....... .......... ..........
AG40     .......... .......... .......... .......... ..........
AG42     .......... .......... .......... .......... ..........
AG43     .......... .......... .......... .......... ..........
AG1M     .......... .......... .......... .......... ..........
AG2M     .......... .......... .......... .......... ..........
AG3M     .......... .......... ..C....... .......... ..........
AG4M     .......... .......... ..C....... .......... ..........
```

```
                                   410        420        430        440        450
       ....|....| ....|....| ....|....| ....|....| ....|....|
V00654 CTAGGCAGGG ATCCCTCTTC TCGCTCCGGG CCCATAAACC GTGGGGGTCG
AG01   .......... .......... .......... .......... ..........
AG02   .......... .......... .......... .......... ..........
AG04   .......... .......... .......... .......... ..........
AG05   .......... .......... .......... .......... ..........
AG06   .......... .......... .......... .......... ..........
AG07   .......... .......... .......... .......... ..........
AG08   .......... .......... .......... .......... ..........
AG09   .......... .......... .......... .......... ..........
AG10   .......... .......... .......... .......... ..........
AG11   .......... .......... .......... .......... ..........
AG13   .......... .......... .......... .......... ..........
AG14   .......... .......... .......... .......... ..........
AG15   .......... .......... .......... .......... ..........
AG16   ...A...A.. .......... .......... .......... ..........
AG18   .......... .......... .......... .......... ..........
AG20   .......... .......... .......... .......... ..........
AG21   .......... .......... .......... .......... ..........
AG22   .......... .......... .......... .......... ..........
AG24   .......... .......... .......... .......... ..........
AG25   .......... .......... .......... .......... ..........
AG26   .......... .......... .......... .......... ..........
AG27   .......... .......... .......... .......... ..........
AG28   .......... .......... .......... .......... ..........
AG29   .......... .......... .......... .......... ..........
AG30   .......... .......... .......... .......... ..........
AG31   .......... .......... .......... .......... ..........
AG40   .......... .......... .......... .......... ..........
AG42   ..G....... .......... .......... .......... ..........
AG43   .......... .......... .......... .......... ..........
AG1M   .......... .......... .......... .......... ..........
AG2M   ..G....... .......... .......... .......... ..........
AG3M   .......... .......... .......... .......... ..........
AG4M   .......... .......... .......... .......... ..........

                                   460        470        480        490        500
       ....|....| ....|....| ....|....| ....|....| ....|....|
V00654 CTATCCAATG AATTTTACCA GGCATCTGGT TCTTTCTTCA GGGCCATCTC
AG01   .......... .......... .......... .......... ..........
AG02   .......... .......... .......... .......... ..........
AG04   .......... .......... .......... .......... ..........
AG05   .......... .......... .......... .......... ..........
AG06   .......... .......... .......... .......... ..........
AG07   ....T..... .......... .......... .......... ..........
AG08   .......... .......... .......... .......... ..........
AG09   .......... .......... .......... .......... ..........
AG10   .......... .......... .......... .......... ..........
AG11   .......... .......... .......... .......... ..........
AG13   .......... .......... .......... .......... ..........
AG14   .......... .......... .......... .......... ..........
AG15   .......... .......... .......... .......... ..........
AG16   .......... ..C....... .......... .......... ..........
AG18   .......... .......... .......... .......... ..........
AG20   .......... .......... .......... .......... ..........
AG21   .......... .......... .......... .......... ..........
AG22   .......... .......... .......... .......... ..........
AG24   .......... .......... .......... .......... ..........
AG25   .......... .......... .......... .......... ..........
AG26   .......... .......... .......... .......... ..........
AG27   .......... .......... .......... .......... ..........
AG28   .......... .......... .......... .......... ..........
AG29   .......... .......... .......... .......... ..........
AG30   .......... .......... .......... .......... ..........
AG31   .......... .......... .......... .......... ..........
AG40   .......... ..C....... .......... .......... ..........
AG42   .......... .......... .A........ .......... ..........
AG43   .......... ..C....... .......... .......... ..........
AG1M   .......... .......... .A........ .......... ..........
AG2M   .......... .......... .A........ .......... ..........
AG3M   .......... .......... .......... .......... ..........
AG4M   .......... .......... .......... .......... ..........
```

```
                 510        520        530        540        550
         ....|....| ....|....| ....|....| ....|....| ....|....|
V00654   ATCTAAAACG GTCCATTCTT TCCTCTTAAA TAAGACATCT CGATGGACTA
AG01     .......... .......... .......... .......... ..........
AG02     .......... .......... .......... .......... ..........
AG04     .......... .......... .......... .......... ..........
AG05     .......... .......... .......... .......... ..........
AG06     .......... .......... .......... .......... ..........
AG07     .......... .......... .......... .......... ..........
AG08     .......... .......... .......... .......... ..........
AG09     .......... .......... .......... .......... ..........
AG10     .......... .......... .......... .......... ..........
AG11     .......... .......... .......... .......... ..........
AG13     .......... .......... .......... .......... ..........
AG14     .......... .......... .......... .......... ..........
AG15     .......... .......... .......... .......... ..........
AG16     .......... .......... .......... .......... ..........
AG18     ........T. .......... .......... .......... ..........
AG20     .......... .......... .......... .......... ..........
AG21     .......... .......... .......... .......... ..........
AG22     .......... .......... .......... .......... ..........
AG24     .......... .......... .......... .......... ..........
AG25     .......... .......... .......... .......... ..........
AG26     .......... .......... .......... .......... ..........
AG27     .......... .......... .......... .......... ..........
AG28     .......... .......... .......... .......... ..........
AG29     ........T. .......... .......... .......... ..........
AG30     .......... .......... .......... .......... ..........
AG31     .......... .......... .......... .......... ..........
AG40     .......... .......... .......... .......... ..........
AG42     .......... .......... .......... .......... ..........
AG43     .......... .......... .......... .......... ..........
AG1M     .......... .......... .......... .......... ..........
AG2M     .......... .......... .......... .......... ..........
AG3M     .......... .......... .......... .......... ..........
AG4M     .......... .......... .......... .......... ..........

                 560        570        580        590        600
         ....|....| ....|....| ....|....| ....|....| ....|....|
V00654   ATGGCTAATC AGCCCATGCT CACACATAAC TGTGCTGTCA TACATTTGGT
AG01     .......... .......... .......... .......... ..........
AG02     .......... .......... .......... .......... ..........
AG04     .......... .......... .......... .......... ..........
AG05     .......... .......... .......... .......... ..........
AG06     .......... .......... .......... .......... ..........
AG07     .......... .......... .......... .......... ..........
AG08     .......... .......... .......... .......... ..........
AG09     .......... .......... .......... .......... ..........
AG10     .......... .......... .......... .......... ..........
AG11     .......... .......... .......... .......... ..........
AG13     .......... .......... .......... .......... ..........
AG14     .......... .......... .......... .......... ..........
AG15     .......... .......... .......... .......... ..........
AG16     .......... .......... .......... .......... ..........
AG18     .......... .......... .......... .......... ..........
AG20     .......... .......... .......... .......... ..........
AG21     .......... .......... .......... .......... ..........
AG22     .......... .......... .......... .......... ..........
AG24     .......... .......... .......... .......... ..........
AG25     .......... .......... .......... .......... ..........
AG26     .......... .......... .......... .......... ..........
AG27     .......... .......... .......... .......... ..........
AG28     .......... .......... .......... .......... ..........
AG29     .......... .......... .......... .......... ..........
AG30     .......... .......... .......... .......... ..........
AG31     .......... .......... .......... .......... ..........
AG40     .......... .......... .......... .......... ..........
AG42     .......... .......... .......... .......... ..........
AG43     .......... .......... .......... .......... ..........
AG1M     .......... .......... .......... .......... ..........
AG2M     .......... .......... .......... .......... ..........
AG3M     .......... .......... .......... .......... ..........
AG4M     .......... .......... .......... .......... ..........
```

```
                   610        620        630        640        650
          ....|....| ....|....| ....|....| ....|....| ....|....|
V00654    ATTTTTTTAT TTTGGGGGAT GCTTGGACTC AGCTATGGCC GTCAAAGGCC
AG01      .......... .......... .......... .......... ..........
AG02      .......... .......... .......... .......... ..........
AG04      .......... .......... .......... .......... ..........
AG05      .......... .......... .......... .......... ..........
AG06      .......... .......... .......... .......... ..........
AG07      .......... .......... .......... .......... ..........
AG08      .......... .......... .......... .......... ..........
AG09      .......... .......... .......... .......... ..........
AG10      .......... .......... .......... .......... ..........
AG11      .......... .......... .......... .......... ..........
AG13      .......... .......... .......... .......... ..........
AG14      .......... .......... .......... .......... ..........
AG15      .......... .......... .......... .......... ..........
AG16      .......... .......... .......... .......... ..........
AG18      .......... .......... .......... .......... ..........
AG20      .......... .......... .......... .......... ..........
AG21      .......... .......... .......... .......... ..........
AG22      .......... .......... .......... .......... ..........
AG24      .......... .......... .......... .......... ..........
AG25      .......... .......... .......... .......... ..........
AG26      .......... .......... .......... .......... ..........
AG27      .......... .......... .......... .......... ..........
AG28      .......... .......... .......... .......... ..........
AG29      .......... .......... .......... .......... ..........
AG30      .......... .......... .......... .......... ..........
AG31      .......... .......... .......... .......... ..........
AG40      .......... .......... .......... .......... ..........
AG42      .......... .......... .......... .......... ..........
AG43      .......... .......... .......... .......... ..........
AG1M      .......... .......... .......... .......... ..........
AG2M      .......... .......... .......... .......... ..........
AG3M      .......... .......... .......... .......... ..........
AG4M      .......... .......... .......... .......... ..........

                   660        670        680        690        700
          ....|....| ....|....| ....|....| ....|....| ....|....|
V00654    CTGACCCGGA GCATCTATTG TAGCTGGACT TAACTGCATC TTGAGCACCA
AG01      .......... .......... .......... .......... ..........
AG02      .......... .......... .......... .......... ..........
AG04      .......... .......... .......... .......... ..........
AG05      .......... .......... .......... .......... ..........
AG06      .......... .......... .......... .......... ..........
AG07      .......... .......... .......... .......... ..........
AG08      .......... .......... .......... .......... ..........
AG09      .......... .......... .......... .......... ..........
AG10      .......... .......... .......... .......... ..........
AG11      .......... .......... .......... .......... ..........
AG13      .......... .......... .......... .......... ..........
AG14      .......... .......... .......... .......... ..........
AG15      .......... .......... .......... .......... ..........
AG16      .......... .......... .......... .......... ..........
AG18      .......... .......... .......... .......... ..........
AG20      .......... .......... .......... .......... ..........
AG21      .......... .......... .......... .......... ..........
AG22      .......... .......... .......... .......... ..........
AG24      .......... .......... .......... .......... ..........
AG25      .......... .......... .......... .......... ..........
AG26      .......... .......... .......... .......... ..........
AG27      .......... .......... .......... .......... ..........
AG28      .......... .......... .......... .......... ..........
AG29      .......... .......... .......... .......... ..........
AG30      .......... .......... .......... .......... ..........
AG31      .......... .......... .......... .......... ..........
AG40      .......... .......... .......... .......... ..........
AG42      .......... .......... .......... .......... ..........
AG43      .......... .......... .......... .......... ..........
AG1M      .......... .......... .......... .......... ..........
AG2M      .......... .......... .......... .......... ..........
AG3M      .......... .......... .......... .......... ..........
AG4M      .......... .......... .......... .......... ..........
```

```
                    710        720        730        740        750
           ....|....| ....|....| ....|....| ....|....| ....|....|
V00654     GCATAATGAT AAGCATGGAC ATTACAGTCA ATGGTCACAG GACATAAATT
AG01       .......... .....G.... .......... .......... ..........
AG02       .......... .....G.... .......... .......... ..........
AG04       .......... .....G.... .......... .......... ..........
AG05       .......... .....G.... .......... .......... ..........
AG06       .......... .....G.... .......... .......... ..........
AG07       .......... .....G.... .......... .......... ..........
AG08       .......... .......... .......... .......... ..........
AG09       .......... .......... .......... .......... ..........
AG10       .......... .....G.... .......... .......... ..........
AG11       .......... .....G.... .......... .......... ..........
AG13       .......... .......... .......... .......... ..........
AG14       .......... .......... .......... .......... ..........
AG15       .......... .....G.... .......... .......... ..........
AG16       .......... .....G.... .......... .......... ..........
AG18       .......... .....G..G. .......... .......... ..........
AG20       .......... .......... .......... .......... ..........
AG21       .......... .....G.... .......... .......... ..........
AG22       .......... .......... .......... .......... ..........
AG24       .......... .......... .......... .......... ..........
AG25       .......... .......... .......... .......... ..........
AG26       .......... .......... .......... .......... ..........
AG27       .......... ........G. .......... .......... ..........
AG28       .......... ........G. .......... .......... ..........
AG29       .......... .....G..G. .......... .......... ..........
AG30       .......... .....G.... .......... .......... ..........
AG31       .......... .......... .......... .......... ..........
AG40       .......... .....G.... .......... .......... ..........
AG42       .......... .......... .......... .......... ..........
AG43       .......... .....G.... .......... .......... ..........
AG1M       .......... .....G.... .......... .......... ..........
AG2M       .......... .....G.... .......... .......... ..........
AG3M       .......... .......... .......... .......... ..........
AG4M       .......... .......... .......... .......... ..........

                    760        770        780        790        800
           ....|....| ....|....| ....|....| ....|....| ....|....|
V00654     ATATTATATA TCCCCCC-TT CATAAAAATT TCCCCCTTAA ATATCTACCA
AG01       .......... .......C.. .......... .......... ..........
AG02       .......... .......C.. .......... .......... ..........
AG04       .......... .......C.. .......... .......... ..........
AG05       .......... .......C.. .......... .......... ..........
AG06       .......... .......C.. .......... .......... ..........
AG07       .......... .......C.. .......... .......... ..........
AG08       .......... .......C.. .......... .......... ..........
AG09       .......... .......C.. .......... .......... ..........
AG10       .......... .......C.. .......... .......... ..........
AG11       .......... .......C.. .......... .......... ..........
AG13       .......... .......C.. .......... .......... ..........
AG14       .......... .......C.. .......... .......... ..........
AG15       .......... .......C.. .......... .......... ..........
AG16       .......... .......-.. .......... .......... ..........
AG18       .......... .......C.. .......... .......... ..........
AG20       .......... .......C.. .......... .......... ..........
AG21       .......... .......C.. .......... .......... ..........
AG22       .......... .......C.. .......... .......... ..........
AG24       .......... .......C.. .......... .......... ..........
AG25       .......... .......C.. .......... .......... ..........
AG26       .......... .......C.. .......... .......... ..........
AG27       .......... .......C.. .......... .......... ..........
AG28       .......... .......C.. .......... .......... ..........
AG29       .......... .......C.. .......... .......... ..........
AG30       .......... .......C.. .......... .......... ..........
AG31       .......... .......C.. .......... .......... ..........
AG40       .......... .......-.. .......... .......... ..........
AG42       .......... .......C.. .......... .......... ..........
AG43       .......... .......-.. .......... .......... ..........
AG1M       .......... .......C.. .......... .......... ..........
AG2M       .......... .......C.. .......... .......... ..........
AG3M       .......... .......C.. .......... .......... ..........
AG4M       .......... .......C.. .......... .......... ..........
```

```
                 810        820        830        840        850
         ....|....| ....|....| ....|....| ....|....| ....|....|
V00654   CCACTTTTAA CAGACTTTTC CCTAGATACT TATTTAAATT TTTCACGCTT
AG01     .......... .......... .......... .......... ..........
AG02     .......... .......... .......... .......... ..........
AG04     .......... .......... .......... .......... ..........
AG05     .......... .......... .......... .......... ..........
AG06     .......... .......... .......... .......... ..........
AG07     .......... .......... .......... .......... ..........
AG08     .......... .......... .......... .......... ..........
AG09     .......... .......... .......... .......... ..........
AG10     .......... .......... .......... .......... ..........
AG11     .......... .......... .......... .......... ..........
AG13     .......... .......... .......... .......... ..........
AG14     .......... .......... .......... .......... ..........
AG15     .......... .......... .......... .......... ..........
AG16     .......... .......... .......... .......... ..........
AG18     .......... .......... .......... .......... ..........
AG20     .......... .......... .......... .......... ..........
AG21     .......... .......... .......... .......... ..........
AG22     .......... .......... .......... .......... ..........
AG24     .......... .......... .......... .......... ..........
AG25     .......... .......... .......... .......... ..........
AG26     .......... .......... .......... .......... ..........
AG27     .......... .......... .......... .......... ..........
AG28     .......... .......... .......... .......... ..........
AG29     .......... .......... .......... .......... ..........
AG30     .......... .......... .......... .......... ..........
AG31     .......... .......... .......... .......... ..........
AG40     .......... .......... .......... .......... ..........
AG42     .......... .......... .......... .......... ..........
AG43     .......... .......... .......... .......... ..........
AG1M     .......... .......... .......... .......... ..........
AG2M     .......... .......... .......... .......... ..........
AG3M     .......... .......... .......... .......... ..........
AG4M     .......... .......... .......... .......... ..........

                 860        870        880        890        900
         ....|....| ....|....| ....|....| ....|....| ....|....|
V00654   TCAATACTCA ATTTAGCACT CCAAACAAAG TCAATATATA AACGCAGGCC
AG01     .......... .......... .......... .......... ..........
AG02     .......... .......... .......... .......... ..........
AG04     .......... .......... .......... .......... ..........
AG05     .......... .......... .......... .......... ..........
AG06     .......... .......... .......... .......... ..........
AG07     .......... .......... .......... .......... ..........
AG08     .......... .......... .......... .......... ..........
AG09     .......... .......... .......... .......... ..........
AG10     .......... .......... .......... .......... ..........
AG11     .......... .......... .......... .......... ..........
AG13     .......... .......... .......... .......... ..........
AG14     .......... .......... .......... .......... ..........
AG15     .......... .......... .......... .......... ..........
AG16     .......... .......... .......... .......... ..........
AG18     .......... .......... .......... .......... ..........
AG20     .......... .......... .......... .......... ..........
AG21     .......... .......... .......... .......... ..........
AG22     .......... .......... .......... .......... ..........
AG24     .......... .......... .......... .......... ..........
AG25     .......... .......... .......... .......... ..........
AG26     .......... .......... .......... .......... ..........
AG27     .......... .......... .......... .......... ..........
AG28     .......... .......... .......... .......... ..........
AG29     .......... .......... .......... .......... ..........
AG30     .......... .......... .......... .......... ..........
AG31     .......... .......... .......... .......... ..........
AG40     .......... .......... .......... .......... ..........
AG42     .......... .......... .......... .......... .......C..
AG43     .......... .......... .......... .......... ..........
AG1M     .......... .......... .......... .......... ..........
AG2M     .......... .......... .......... .......... ..........
AG3M     .......... .......... .......... .......... ..........
AG4M     .......... .......... .......... .......... ..........
```

```
          . . . . | . . . .
V00654    C C C C C C C C C
AG01      . . . . . . . . .
AG02      . . . . . . . . .
AG04      . . . . . . . . .
AG05      . . . . . . . . .
AG06      . . . . . . . . .
AG07      . . . . . . . . .
AG08      . . . . . . . . .
AG09      . . . . . . . . .
AG10      . . . . . . . . .
AG11      . . . . . . . . .
AG13      . . . . . . . . .
AG14      . . . . . . . . .
AG15      . . . . . . . . .
AG16      . . . . . . . . .
AG18      . . . . . . . . .
AG20      . . . . . . . . .
AG21      . . . . . . . . .
AG22      . . . . . . . . .
AG24      . . . . . . . . .
AG25      . . . . . . . . .
AG26      . . . . . . . . .
AG27      . . . . . . . . .
AG28      . . . . . . . . .
AG29      . . . . . . . . .
AG30      . . . . . . . . .
AG31      . . . . . . . . .
AG40      . . . . . . . . .
AG42      . . . . . . . . .
AG43      . . . . . . . . .
AG1M      . . . . . . . . .
AG2M      . . . . . . . . .
AG3M      . . . . . . . . .
AG4M      . . . . . . . . .
```
